# Are People Altruistic When Making Socially Responsible Investments? Evidence From a tDCS Study

**DOI:** 10.3389/fnins.2021.704537

**Published:** 2021-08-18

**Authors:** Xiaolan Yang, Wenting Meng, Shu Chen, Mei Gao, Jian Zhang

**Affiliations:** ^1^School of Business and Management, Key Laboratory of Applied Brain and Cognitive Sciences, Shanghai International Studies University, Shanghai, China; ^2^Center for Economic Behavior and Decision-Making (CEBD), Zhejiang University of Finance and Economics, Hangzhou, China

**Keywords:** socially responsible investment, altruism, motivation, right temporoparietal junction, transcranial direct current stimulation

## Abstract

Socially responsible investment (SRI) is an emerging philosophy that integrates social and environmental impacts into investment considerations, and it has gradually developed into an important form of investment. Previous studies have shown that both financial and non-financial motivations account for SRI behaviors, but it is unclear whether the non-financial motive to adopt SRI derives from investors’ altruism. This study uses neuroscientific techniques to explore the role of altruism in SRI decision-making. Given that existing evidence has supported the involvement of the right temporoparietal junction (rTPJ) in altruism and altruistic behaviors, we used transcranial direct current stimulation (tDCS) to temporarily modulate activity in the rTPJ and tested its effect on charitable donations and SRI behaviors. We found that anodal stimulation increased the subjects’ donations, while cathodal stimulation decreased them, suggesting that tDCS changed the subjects’ levels of altruism. More importantly, anodal stimulation enhanced the subjects’ willingness to make SRIs, while cathodal stimulation did not have a significant impact. These findings indicate that altruism plays an important role in SRI decision-making. Furthermore, cathodal stimulation changed the subjects’ perceived effectiveness of charitable donation but not that of socially responsible fund. This result may help explain the inconsistent effects of cathodal stimulation on charitable donations and SRI behaviors. The main contribution of our study lies in its pioneering application of tDCS to conduct research on SRI behaviors and provision of neuroscientific evidence regarding the role of altruism in SRI decision-making.

## Introduction

Socially responsible investment (SRI) is an investment discipline that adds concerns about social or environmental issues as a determinant of investment portfolio construction or investment activities in the consideration of investment risks and returns (Sparkes and Cowton, 2004; Sparkes, 2008). As an emerging investment philosophy, SRI has been favored by an increasing number of investors in recent years, and it has gradually developed into an important form of investment (Eurosif, 2020). Notably, SRI has expanded rapidly throughout the COVID-19 pandemic. For instance, in the second quarter of 2020, global sustainable fund inflows increased by 72%, with assets under management exceeding US$1 trillion for the first time (Morningstar, 2020). Therefore, it is important to better understand SRI behaviors and, in particular, the psychological motivations of SRI investors.

Studies have explored the motivations behind SRI. Some studies show that SRI is driven by financial motives for higher returns or lower risks. For instance, Jansson and Biel (2011) found that the main motivation for investors to engage in SRI lies in a belief that socially responsible assets can bring higher investment returns. From a questionnaire survey, Glac (2009) found that when making investment decisions, financial considerations are usually more prominent than social considerations; thus, investors are usually unwilling to sacrifice financial returns to follow their beliefs. Døskeland and Pedersen (2016) and Riedl and Smeets (2017) noted that once investors perceive the expected returns from socially responsible assets to be poor or lower than those from traditional assets, their willingness to make SRIs will decrease. In addition, people believe that socially responsible companies usually face fewer reputational and litigation risks or that, at the very least, they will achieve less risk under the same financial benefits (Beal et al., 2005; Renneboog et al., 2008). From a questionnaire survey, Dorfleitner and Utz (2014) found that expectations of returns and risks significantly affect investors’ SRI behaviors and willingness to sacrifice returns. Empirical studies have also found that the financial performance of portfolios with high levels of social responsibility is generally better with regard to returns and risks (Orlitzky and Benjamin, 2001; Derwall et al., 2004).

Other studies have provided evidence that SRI is also driven by non-financial motives, which are believed to derive from considerations of the impact of investment decisions on social interests. Lewis and Mackenzie (2000) found that investors generally face a dilemma between pursuing morality and pursuing their financial interests. Statman (2004) and Nilsson (2009) found that investors show significant individual differences in their evaluations of financial returns and social responsibility; investors with high levels of social responsibility are willing to sacrifice more financial returns for their own moral pursuits. Hartzmark and Sussman (2019) observed investment decisions on traditional assets and socially responsible assets at different return levels and found that investors are willing to sacrifice their own investment returns for SRIs. Bonnefon et al. (2019) found that the prosocial preferences of investors are positively correlated with SRI behavior. Wins and Zwergel (2016) and Brodback et al. (2019) used questionnaires to study the personal values of investors and found that their altruistic values significantly impact their SRI behaviors. In particular, when investors believe that their investment behaviors can play a positive role in society, they will be more willing to make SRIs.

Many of the above studies finding that SRI is partly driven by non-financial motives attributed these motives or directly refer to such motives as prosocial preferences, or more specifically, altruism. Theories of prosocial preferences are based on the notion that people care about the well-being of others (Charness and Rabin, 2002; Meier, 2007). A crucial type of prosocial preference is altruism, and being altruistic means that a person’s utility increases with the well-being of other people (Fehr and Schmidt, 2006). Nevertheless, some other factors may also account for investors’ non-financial motives observed in the real world, such as those of reputational concern and social conformity. Even in an experimental environment, subjects may also unconsciously integrate their real-world experiences into investment tasks. Therefore, more evidence is needed regarding whether altruism plays an important role in SRI decision-making.

Our study uses neuroscientific methods to explore the role of altruism in SRI decision-making. Previous neuroscientific studies have found that the temporoparietal junction (TPJ) plays a key role in altruism and altruistic behaviors. Some studies have found that enhancing activity in the TPJ will increase the empathy and altruistic behaviors of individuals (Jeurissen et al., 2014; van der Meulen et al., 2016). Other studies have found that subjects who are willing to allocate more money to others in a dictator game show stronger activity in their TPJ, especially in the right temporoparietal junction (rTPJ) (Hutcherson et al., 2015; Strombach et al., 2015; Park et al., 2017). Recent studies have also used closer-to-life altruistic tasks to measure subjects’ altruistic preferences by asking them to allocate funds to themselves or charities. Hare et al. (2010) and Tusche et al. (2016) found that subjects who donated more to charities showed higher activity in the rTPJ. Using transcranial direct current stimulation (tDCS), Li et al. (2020) found that those who received anodal stimulation increased their donations to charities.

Evidence also indicates that the functional contribution of the rTPJ to altruism lies in signaling conflicts between moral and material interests. Morishima et al. (2012) found that activity in the rTPJ depends on the cost of altruistic behavior. When the cost of altruism is low, activity in the rTPJ is positively correlated with altruistic behavior. However, when self-interested behavior conflicts with altruistic behavior, this will lead to a decrease in activity in the rTPJ. Obeso et al. (2018) further showed that the rTPJ is involved in handling moral-material conflicts involved in donation behavior. After disrupting the rTPJ using transcranial magnetic stimulation, subjects showed reduced monetary self-interest and donated significantly more than the control group.

This study used tDCS to temporarily modulate activity in the rTPJ and tested how different stimulation modes affected subjects’ donation and SRI behaviors. Based on existing evidence, our hypotheses are as follows. First, modulating activity in the rTPJ using tDCS will alter subjects’ processing of moral-material conflicts, thus changing subjects’ donation behaviors. Second and more importantly, if altruism does play an important role in SRI decision-making, then changes in subjects’ processing of moral-material conflicts will also lead to changes in their SRI behaviors. More specifically, we hypothesize that increasing activity in the rTPJ will increase subjects’ donation and SRI behaviors, while decreasing activity in the right rTPJ will decrease their donation and SRI behaviors. By modulating activity in the rTPJ, we tried to disentangle the motive of altruism and other possible non-financial motives and to see if the process of SRI decision-making does involve altruistic considerations.

## Materials and Methods

### Subjects

A total of 96 subjects (24 males and 72 females; mean age: 21.23 years, ranging from 18 to 28 years) were recruited to participate in our experiment. All of the subjects were students at Shanghai International Studies University, and they were randomly assigned to receive anodal (*n* = 32; males: 8, females: 24; mean age: 21.21), cathodal (*n* = 32; males: 8, females: 24; mean age: 21.21), or sham stimulation (*n* = 32; males: 8, females: 24; mean age: 21.25). All subjects were right-handed, and all of them reported having no history of mental illness or neurological disease and having no experience with tDCS or investment tasks. Before participating in the experiment, the subjects were required to sign a written informed consent form to receive tDCS. The experiment was conducted in the Key Laboratory of Applied Brain and Cognitive Sciences of Shanghai International Studies University, and the experimental scheme was approved by the ethics committee of the laboratory. The whole experiment lasted approximately 1 h, and the subjects received, on average, 60 RMB yuan (approximately $9.17) as compensation. No side effects, such as scalp pain or headache, were reported after the experiment.

### Transcranial Direct Current Stimulation

**FIGURE 1:**
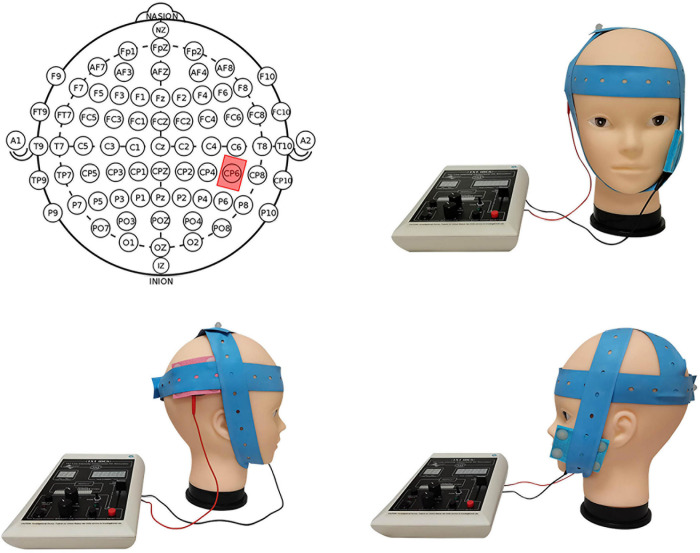
Schematic and locations of the electrodes applied under the anodal stimulation mode.

Transcranial direct current stimulation (tDCS) is a non-invasive form of brain stimulation technology. The stimulation equipment used was developed by Soterix Medical Inc. (New York, United States) and used two saline-soaked sponge electrodes (size: 5 cm × 7 cm) to generate a weak current in the target brain area of the subjects. Figure 1 shows how the electrodes were placed under anodal stimulation conditions. According to the International 10/20 EEG Positioning System (Jasper, 1958), we aimed to place the center of the anodal electrode over CP6 (Jurcak et al., 2007; Koessler et al., 2009), and the cathodal electrode was placed on the subject’s opposite (left) cheek (Berryhill and Jones, 2012; Tseng et al., 2012; Mai et al., 2016). Under cathodal stimulation conditions, we aimed to place the center of the cathodal electrode over CP6, and the anodal electrode was placed on the subject’s left cheek. The sham stimulation conditions randomly adopted the electrode placement of either anodal or cathodal stimulation. The stimulation delivered a constant current of 1.5 mA lasting 20 min to induce changes in the excitability of the cerebral cortex of the target area without causing any physiological harm to the subjects. According to previous studies, the anodal electrode enhances the excitability of the cortex, and the cathodal electrode inhibits the excitability of the cortex (Nitsche and Paulus, 2000). For sham stimulation, the current was delivered for only 30 s, and this method has been proven reliable by previous studies (Gandiga et al., 2006).

### Experimental Design

Our experiment involved the following four tasks performed in fixed order: a charity donation task, simulated SRI task, real SRI task, and risk preference measurement task. Each of the first three tasks was set up with a “personal wallet” and a “charity wallet.” The personal wallet contained the payoffs received by the subject from the task, and the charity wallet contained the charitable donation generated by the task. We chose the Alipay charitable platform as the recipient of the donations since this platform covers a wide range of charity projects (i.e., education assistance, poverty alleviation, disaster relief, medical assistance, and environmental protection) and is held in high esteem in China. Anyone can make online donations easily on this platform through electronic payments.

#### Donation Task

The donation task is a modified version of the dictator game that is usually used to test altruism (Forsythe et al., 1994; Eckel and Grossman, 1996). In the task, the subjects were given a sum of 50 yuan and had to decide how much to donate to charity. The amount donated to charity was transferred to the charity wallet, and the remaining amount was allocated to the subject’s personal wallet. The more money the subject donated to charity, the higher his/her level of altruism was.

#### Simulated SRI Task

The simulated SRI task was designed based on Bonnefon et al. (2019) and Brodback et al. (2020), and we integrated and modified their tasks to the purposes of our research. In our task, the subjects were given 50 yuan and were asked to make bids for an ordinary asset and a socially responsible asset. Both assets had a 50% probability of yielding a return of 40 yuan and a 50% probability of yielding only 10 yuan for the subject’s personal wallet. However, the socially responsible asset would also donate an additional 10 yuan to charity (with the amount in the charity wallet increasing by 10 yuan without changing the amount in the personal wallet) if it was purchased. The subjects were asked to report the highest prices they were willing to pay for the two assets (minimum: 0, maximum: 50). The subjects only made two decisions for this task: a bid for the ordinary asset and then a bid for the socially responsible asset with a fixed order. To avoid the wealth effect, the computer randomly selected one asset to provide payment for this task. To incentivize the subjects to disclose their real evaluations of the assets, we adopted the Becker-DeGroot-Marschak (BDM) bidding mechanism of Becker et al. (1964). This mechanism can ensure that for a rational subject, the optimal choice is to report his/her true willingness to pay. The operation of the mechanism was set as follows: The asset price was randomly generated in the interval of [0,50]. If the subject’s bid was lower than the random price, the asset could not be purchased. If the subject’s bid was equal to or higher than the random price, the asset would be successfully purchased at the random price. Based on the design of the task, a higher bid could be regarded as a greater willingness to invest in the asset. In addition, we could offset the impact of financial motives by calculating the differences between the subjects’ bids for the socially responsible asset and the ordinary asset since both assets have the same levels of risks and returns. In other words, the difference between the bids for the two assets could reflect the subjects’ non-financial motives to engage in SRI.

#### Real SRI Task

The real SRI task involved investment decision-making with regard to a real socially responsible fund. For the task, the subjects were given 50 yuan and were asked to make a bid (minimum: 0, maximum: 50) for a real socially responsible fund, the Xingquan Social Responsibility Mix Fund. This fund is a publicly offered socially responsible fund in China. While pursuing returns, the fund also emphasizes the performance of listed companies in terms of sustainable development, law, and moral responsibility. The fund can be easily purchased and sold through a mobile app, and the minimum capital requirement is as low as 10 yuan. To incentivize the subjects to disclose their real evaluations of the fund, the task also applied the BDM bidding mechanism, as in the simulated SRI task. If the subject’s bid was equal to or higher than the randomly generated price, the investment was successful, and a real share of the fund worth 50 yuan (at that moment) could be obtained at the generated random price. The experimenter helped the subjects purchase the corresponding share of the fund through the app on their own mobile phones when the experiment was over. If the subject’s bid was lower than the randomly generated price, the fund was not bought, and the subject retained 50 RMB yuan. Similarly, the subject’s bid for the fund reflected his/her willingness to invest in real socially responsible funds.

#### Risk Preference Measurement Task

Risk preference plays an important role in investment decision-making. Therefore, we also measured the subjects’ risk preferences to explore whether the effects of stimulation modes on subjects’ investment behaviors were due to changes in their risk preferences. The risk preference measurement task followed the method of Falk et al. (2018) to assess the subjects’ risk preferences. The task consisted of two parts. For the first part, the subjects were asked to rate their own preference for risk on a 10-point scale (i.e., self-rated risk level). The second part involved 5 multiple-choice questions on risk drawn from a pool of 31 multiple-choice questions. Each question in the question bank had two options, A and B, where A was “50% likely to receive 300 yuan, 50% likely to receive 0 yuan” and B was “a fixed reward of X yuan” (where X changes in different questions). For each question displayed, the subjects needed to choose the preferred option, and their choices determined the value of X included in the next question displayed. A staircase risk level could be obtained based on the subjects’ answers to the 5 questions. Based on the results of the two parts, each subject’s level of risk preference could be calculated.

### Experimental Procedure

**FIGURE 2:**
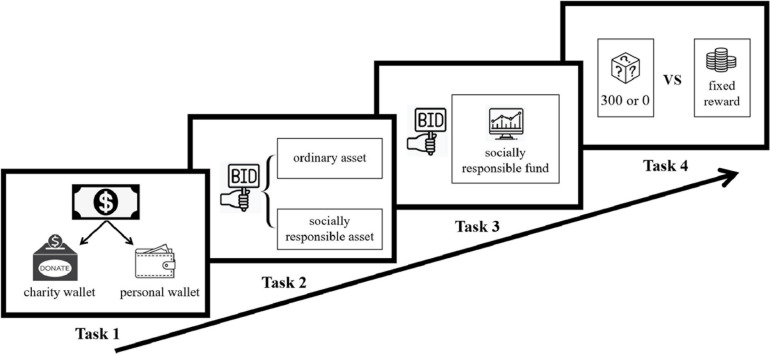
The four sequential tasks of the experiment. The subjects were asked to complete the charity donation, simulated SRI, real SRI, and risk preference measurement tasks in a fixed order. The subjects generally took approximately 20 min to finish all of these tasks.

The experimental tasks were programmed and implemented using oTree software (Chen et al., 2016). At the beginning of the experiment, the subjects were given tDCS for 20 min, during which time they rested in a chair. When the stimulation was over, the devices were removed from the subjects’ heads. Then, the subjects were asked to perform the four tasks described above in sequence (Figure 2) and were told that at the end of the experiment, the computer would randomly select one of the first three tasks to execute the payment of the experiment (including the subjects’ payoffs and charity donations). In addition, to be consistent with the risk preference measurement task of Falk et al. (2018), we did not pay for this task. After all tasks were completed, the subjects were asked to complete a questionnaire on some control factors such as the perceived effectiveness of charity donation (the extent to which the subjects believe that charity donations can have a positive impact on society); the perceived effectiveness of socially responsible fund (the extent to which the subjects believed that investing in socially responsible funds could have a positive impact on society); the subjects’ return and risk performance evaluations of the Xingquan Social Responsibility Mix Fund; and the subjects’ demographic characteristics in terms of gender, age, educational level, and family income level. Then, the computer randomly chose one of the first three tasks to implement payment for the whole experiment, and only when the simulated or real SRI task was chosen was the random price generated. In other words, the subjects did not know their final payoff until the end of the experiment. The subjects generally took approximately 20 min to complete all of the poststimulation tasks. Finally, the subjects received their payoffs and witnessed the online charity donation executed by the experimenter.

## Results

### Effects of tDCS

**FIGURE 3:**
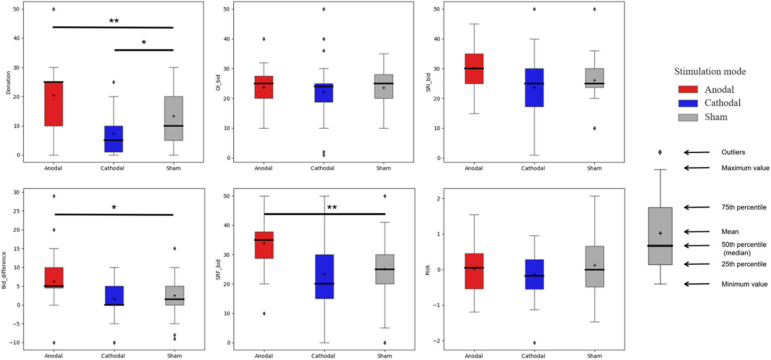
Statistical characteristics of the variables used for each task under different stimulation modes. *Donation* represents the donation amounts given by the subjects for the donation task. *OI_bid* and *SRI_bid* represent the subjects’ bids for the ordinary asset and socially responsible asset, respectively, and *Bid_difference* represents the difference in the subjects’ bids for the two assets (*SRI_bid*-*OI_bid*) for the simulated SRI task. *SRF_bid* represents the subjects’ bids for the socially responsible fund for the real SRI task. *Risk* represents the subjects’ risk preferences under the risk preference measurement task, where the smaller the value is, the less risk-seeking the subject is. Asterisks indicate significant differences (*p < 0.05, **p < 0.01, ***p < 0.001).

Figure 3 summarizes the statistical characteristics of the data obtained from each task under different stimulation modes. We first conducted a one-way ANOVA to test the impact of different stimulation modes on the data for each task. We report the Bonferroni correction results for pairwise comparisons and set the standard for significance at 0.05. Outliers were kept in the analyses because we think that each decision was made by its own logic in our experiment, and it is inappropriate to remove a decision simply because it considerably different from others. Nevertheless, we also ran analyses without outliers, and the conclusions are the same.

To test whether the stimulations changed the subjects’ altruistic preferences, we compared the donation amounts of subjects in different stimulation groups for the donation task and found significant differences (*F*_2_,_93_ = 14.913, *p* < 0.001). Among them, the average donation of the subjects under anodal stimulation was significantly higher than that under sham stimulation (mean: anodal = 20.44, sham = 13.31, *p* = 0.01). Compared to the subjects in the sham stimulation group, the subjects in the cathodal stimulation group were significantly less willing to donate (mean: cathodal = 7.41, sham = 13.31; *p* = 0.046). These results demonstrate that we successfully changed the subjects’ levels of altruism. The results are also consistent with the conclusions of previous studies showing that activity in the rTPJ is positively correlated with the level of altruism (Morishima et al., 2012; Hutcherson et al., 2015; Strombach et al., 2015). In addition, we found a significant effect of stimulation modes on the subjects’ perceived effectiveness of charity donation (*F*_2_,_93_ = 5.102, *p* = 0.008), which was used to measure the extent to which the subjects believed that charity donations could have a positive impact on society. Pairwise comparisons show that cathodal stimulation significantly decreased the subjects’ perceived effectiveness of charity donation, while anodal stimulation did not change it (mean: anodal = 4.47, cathodal = 4.09, sham = 4.53; anodal vs. sham: *p* = 1.000; cathodal vs. sham: *p* = 0.012).

Upon analyzing the asset bids of the subjects for the simulated SRI task, we found, overall, no significant differences in the ordinary asset bids under different stimulation conditions (*F*_2_,_93_ = 0.405, *p* = 0.668). This result indicates that the stimulations did not affect the subjects’ financial motives or willingness to invest in the ordinary asset. In contrast, we found a significant difference in the bids for the socially responsible asset under different stimulation modes (*F*_2_,_93_ = 4.571, *p* = 0.01). The average bid made under anodal and cathodal stimulation conditions was not significantly different from that made under sham stimulation conditions (mean: anodal = 30.16, cathodal = 23.72, sham = 26.13; anodal vs. sham: *p* = 0.192; cathodal vs. sham: *p* = 0.799). Nevertheless, the average bid made in the cathodal stimulation group was significantly lower than that made in the anodal stimulation group (*p* = 0.01). These results preliminarily indicate that the stimulations may have changed the subjects’ evaluations of SRI, but more evidence must be provided.

To further eliminate the impact of financial motives, we subtracted each subject’s ordinary asset bid from his/her socially responsible asset bid, denoting the difference as *Bid_difference*. This variable indicates the strength of the subject’s non-financial motive to engage in SRI. We found significant differences in the *Bid_difference* values of the subjects under different stimulation conditions (*F*_2_,_93_ = 6.366, *p* = 0.003). The average *Bid_difference* of the anodal stimulation group was significantly higher than values for the sham and cathodal stimulation groups (mean: anodal = 6.28, cathodal = 1.59, sham = 2.50; anodal vs. sham: *p* = 0.024; anodal vs. cathodal: *p* = 0.003). However, no significant difference was found between the average *Bid_difference* values of the cathodal and sham stimulation groups (*p* = 1.000). These results further verify that anodal stimulation but not cathodal stimulation changed the subjects’ non-financial motives to engage in SRI.

The subjects’ bids for the socially responsible fund for the real SRI task show similar results. We found significant differences in the bids of different stimulation groups (*F*_2_,_93_ = 8.853, *p* < 0.001). The average bid for the anodal stimulation group was significantly higher than that for the sham and cathodal stimulation groups (mean: anodal = 33.97, cathodal = 23.28, sham = 25.06; anodal vs. sham: *p* = 0.004; anodal vs. cathodal: *p* < 0.001). Consistent with the simulated SRI task, although the subjects of the cathodal stimulation group generally offered lower bids than those of the sham stimulation group, the difference was not significant (*p* = 1.000). Nevertheless, we did not find a significant effect of stimulation modes on the subjects’ perceived effectiveness of socially responsible fund (*F*_2_,_93_ = 0.635, *p* = 0.532), which was used to measure the extent to which the subjects believed that investing in socially responsible funds could have a positive impact on society.

We also compared the risk preferences of the subjects under different stimulation modes to determine whether the stimulations changed their risk preferences. The calculation of the risk preferences was based on Falk et al. (2018). We first standardized the two risk indicators (self-rated risk level and staircase risk level) obtained from the risk preference measurement task and then added them up with different weights (*Risk* = 0.4729985 × staircase risk level + 0.5270015 × self-rated risk level). We found no significant differences in the *Risk* values of the subjects under different stimulation conditions (*p* = 0.36). This result indicates that the stimulations did not affect the subjects’ risk preferences. In other words, the observed effect of stimulation on SRI was not caused by changes in risk preferences.

**FIGURE 4:**
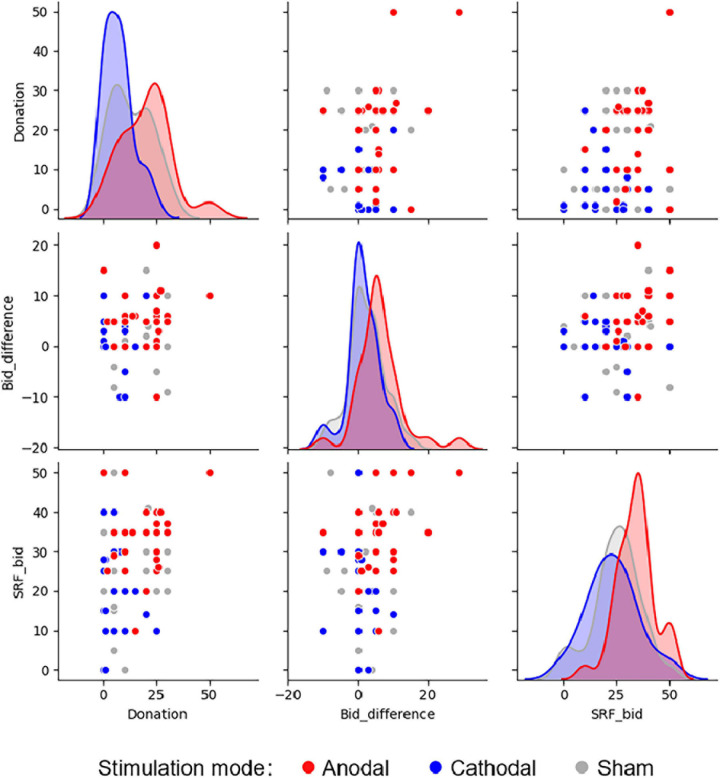
Scatter plots and distribution curves of important variables under different stimulation modes. *Donation* represents the donation amounts given by of the subjects for the donation task. *Bid_difference* represents the difference in the subjects’ bids for the two assets for the simulated SRI task. *SRF_bid* represents the subjects’ bids for the socially responsible fund for the real SRI task. Each dot represents the choice made by one subject.

Figure 4 further shows the scatter plots and distribution curves of *Donation*, *Bid_difference*, and *SRF_bid* for different stimulation modes. We find that the distributions of these variables are generally consistent with the results of the one-way ANOVAs. The distribution curves of *Donation* for the three stimulation groups differ to some extent, indicating that both anodal and cathodal stimulations changed the subjects’ levels of altruism. In contrast, the distribution curves of *Bid_difference* and *SRF_bid* for the cathodal and sham stimulations are more similar, showing that only the anodal stimulation changed the subjects’ non-financial motives to engage in SRI.

### Robustness Tests

Next, we conducted ANCOVAs to test whether the effects of the stimulations were robust when controlling for other factors. We took the stimulation mode as a fixed factor and other related variables as covariates. The results and parameter estimates are shown in Table 1. Again, we report the Bonferroni correction results for pairwise comparisons and set the standard for significance to 0.05. Outliers were also kept in the analyses as described in Section 3.1. We also ran analyses without outliers, and the conclusions were found to be the same.

**TABLE 1:** Results of the ANCOVA models and parameter estimates.

	**(1)**	**(2)**	**(3)**	**(4)**	**(5)**	**(6)**	**(7)**
	***Donation***	***OI_bid***	***SRI_bid***	***SRI_bid***	***Bid_difference***	***SRF_bid***	***Risk***
*Anodal*	2.773** (2.46)	0.044 (1.972)	2.493* (1.927)	3.606*** (1.260)	3.333*** (1.321)	3.138** (2.547)	–0.309 (0.194)
*Cathodal*	–2.686** (2.572)	–0.483 (1.987)	0.453 (2.020)	0.468 (1.318)	0.533 (1.385)	–0.600 (2.572)	–1.121 (0.194)
*PCE_donation*	–1.129 (1.708)		3.715*** (1.351)	3.494*** (0.899)	2.477* (0.926)		
*Risk*		2.708** (1.077)	3.212** (1.062)		1.086 (0.728)	3.547*** (1.367)	
*OI_bid*				11.855*** (0.067)			
*fund_return*						1.055 (1.614)	
*fund_risk*						–1.240 (1.379)	
*PCE_SRF*						1.428 (1.353)	
Gender	–0.189 (2.329)	0.422 (1.870)	–1.074 (1.826)	–2.127* (1.195)	–2.095* (1.251)	–2.632** (2.383)	0.387 (0.184)
Age	–0.393 (0.794)	–0.548 (0.646)	0.838 (0.631)	2.337* (0.407)	2.041* (0.433)	0.452 (0.821)	1.633 (0.063)
Education	0.154 (3.293)	0.009 (2.650)	–0.691 (2.590)	–1.355 (1.687)	–1.108 (1.775)	–1.362 (3.397)	–0.918 (0.260)
Income	0.350 (0.929)	0.319 (0.753)	0.047 (0.736)	–0.167 (0.478)	–0.616 (0.505)	–0.530 (0.959)	1.379 (0.073)
Constant	1.771 (15.662)	2.662** (11.213)	–0.348 (12.617)	–2.740** (8.052)	–2.496* (8.648)	1.319 (16.740)	–1.843 (1.083)
R^2^	0.258	0.095	0.332	0.714	0.278	0.373	0.076
Adjusted R^2^	0.198	0.023	0.271	0.688	0.211	0.299	0.014
F	4.360***	1.314	5.407***	27.195***	4.178***	5.056***	1.228
N	96	96	96	96	96	96	96

*Anodal*/*Cathodal* denotes that the subject received anodal/cathodal stimulation (baseline: sham stimulation). *PCE_donation* represents the subjects’ perceived effectiveness of charity donation. *Fund_return* and *fund_risk* represent the subjects’ return and risk performance evaluations of the Xingquan Social Responsibility Mix Fund, respectively. *PCE_SRF* represents the subjects’ perceived effectiveness of socially responsible fund. *Gender* takes a value of 1 for females and a value of 0 for males. Standard errors are shown in parentheses and asterisks indicate significant differences (* 0.05, ** 0.01, *** 0.001).

For the donation task, we took *Donation* as the dependent variable and the perceived effectiveness of charity donation, gender, age, educational level, and income level as covariates (Model 1). After adding the covariates, we still found a significant effect of the stimulation mode (*F*_2_,_88_ = 14.880, *p* < 0.001, η^2^ = 0.253). The donation amount of the anodal stimulation group was significantly higher than the values for the sham (*p* = 0.02) and cathodal stimulation groups (*p* < 0.001), while the donation amount of the cathodal stimulation group was significantly lower than that of the sham stimulation group (*p* = 0.026). Moreover, we still found a significant effect of the stimulation mode on the subjects’ perceived effectiveness of charity donation after controlling for gender, age, educational level, and income level (*F*_2_,_89_ = 4.886, *p* = 0.010, η^2^ = 0.099). Cathodal stimulation significantly decreased the subjects’ perceived effectiveness of charity donation (*B* = –0.441, *p* = 0.005), while anodal stimulation did not change it (*B* = –0.068, *p* = 0.658).

For the simulated SRI task, we took the subjects’ bids for the ordinary asset as the dependent variable and took risk preferences, gender, age, educational level, and income level as covariates (Model 2). The results show that when the covariates were added, the stimulation mode still had no significant effect on the bid (*F*_2_,_88_ = 0.173, *p* = 0.841, η^2^ = 0.004). However, the impact of risk preferences on the bid was significant (*p* = 0.008). The more risk-seeking the subject was, the more he/she bid for the asset. These results further verify that the stimulations did not affect the subjects’ financial motives.

When taking the subjects’ bids for the socially responsible asset as the dependent variable, we first used risk preferences, the perceived effectiveness of charity donations, gender, age, educational level, and income level as covariates (Model 3). After adding these covariates, we found that the stimulation mode still had a significant impact on the asset bid (*F*_2_,_87_ = 3.527, *p* = 0.034, η^2^ = 0.075). Moreover, compared to those found from the one-way ANOVAs, the differences in the bids of the anodal and sham stimulation groups became more significant (with a decrease in *p* from 0.192 to 0.044). The cathodal stimulation group did not lower the asset bid relative to the sham stimulation group (*p* = 1.000), which is consistent with the results of the one-way ANOVAs. The subjects’ risk preferences and perceived effectiveness of charity donation also had significant impacts on the bid. The stronger risk preferences and perceived effectiveness were, the higher the bid became *(Risk*: *p* = 0.002, *PCE_donation*: *p* < 0.001).

Since the bids for the ordinary asset denote the subjects’ preferences and considerations of risks and returns, they could also be used as a factor in predicting bids for the socially responsible asset. We used the subjects’ bids for the socially responsible asset as the dependent variable and took their bids for the ordinary asset, and the perceived effectiveness of charity donations, gender, age, educational level, and income level as covariates (Model 4). We found that the stimulation mode had a very significant impact on the bids for the socially responsible asset (*F*_2_,_87_ = 7.685, *p* = 0.001, η^2^ = 0.150). The bids of the anodal stimulation group were significantly higher than those of the sham (*p* = 0.002) and cathodal stimulation groups (*p* = 0.009). Consistent with the results of the one-way ANOVAs, we found no significant difference between the cathodal and sham stimulation groups (*p* = 1.000). We also observed that the higher the subjects’ bids for the ordinary asset and the higher the degree of the perceived effectiveness of charity donation became, the higher the bids for the socially responsible asset became (*OI_bid*: *p* < 0.001, *PCE_donation*: *p* = 0.001). In addition, gender and age had a significant impact on asset bids (*gender*: *p* = 0.036, *age*: *p* = 0.022). Bids made by females were lower than those made by males, and for all subjects, the older a subject was, the higher the bid made was.

We also used the subjects’ differences in bids between ordinary and socially responsible assets as the dependent variable and took risk preferences, the perceived effectiveness of charity donations, gender, age, educational level, and income level as covariates (Model 5). Doing so was equivalent to imposing a restriction on Model 4 and fixing the coefficient of *OI_bid* to 1. The results still show significant differences in the asset bids of the different stimulation groups (*F*_2_,_87_ = 6.409, *p* = 0.003, η^2^ = 0.128). This result indicates that different stimulation groups show significant differences in their non-financial motivations to engage in SRI. The subjects who received anodal stimulation show significantly more non-financial motivation to engage in SRI than those who received sham (*p* = 0.004) and cathodal stimulation (*p* = 0.025). However, no significant differences were found between the cathodal and sham stimulation groups. In addition, the greater the perceived effectiveness of charity donation was, the higher the bid was (*p* = 0.015). Gender (females’ bids were lower than males’) and age (older subjects made higher bids) also had significant impacts on asset bids (*gender*: *p* = 0.039, *age*: *p* = 0.044). Notably, the impact of risk preferences on bids was no longer significant (*p* = 0.281), further verifying that the method used to calculate the difference between socially responsible asset bids and ordinary asset bids could effectively offset the influence of financial motives on SRI decision-making.

For the real SRI task, we took the subjects’ bids for the real socially responsible fund as the dependent variable and took risk preferences, the perceived effectiveness of the socially responsible fund, the return and risk performance evaluations of the Xingquan Social Responsibility Mix Fund, gender, age, educational level, and income level as covariates (Model 6). We found that the impact of the stimulation mode on the fund bids to still be very significant (*F*_2_,_85_ = 8.388, *p* < 0.001, η^2^ = 0.164). The fund bids of the anodal stimulation group were significantly higher than those of the sham (*p* = 0.007) and cathodal stimulation groups (*p* = 0.001), while there were no significant differences between the cathodal and sham stimulation groups (*p* = 1.000). This result is consistent with the results of the one-way ANOVAs. In addition, gender (females make lower bids than males) and risk preferences (the stronger risk preferences are, the higher the bid becomes) had significant impacts on the fund bids *(gender*: *p* = 0.01, *risk preference*: *p* = 0.001). The return and risk performance evaluations were not significant, indicating that the subjects’ bids were not relying on their expectations surrounding SRI risks and returns (*fund_return*: *p* = 0.294, *fund_risk*: *p* = 0.219). Moreover, we still did not find a significant effect of the stimulation mode on the subjects’ perceived effectiveness of socially responsible fund after controlling for gender, age, educational level, and income level (*F*_2_,_89_ = 0.592, *p* = 0.555, η^2^ = 0.013).

Finally, to test whether the stimulation modes affected the risk preferences of the subjects, we used risk preferences as the dependent variable and gender, age, educational level, and income level as covariates (Model 7). Again, consistent with the results of the one-way ANOVAs, we found no significant differences in the risk preferences of the subjects under different stimulation conditions (*F*_2_,_89_ = 0.674, *p* = 0.512, η^2^ = 0.015). This result shows that the stimulation modes did not affect the subjects’ risk preferences.

## Discussion

With the rapid development of SRI in recent years, the motivation to make SRIs has become an important topic. Studies have found that SRI is driven by both financial and non-financial motives (Lewis and Mackenzie, 2000; Statman, 2004; Nilsson, 2008). These non-financial motives are usually attributed to altruism, but other factors may also account for these motives, such as reputational concern and social conformity. This study explored whether altruism plays an important role in SRI decision-making. We used tDCS to temporarily modulate activity in the rTPJ and tested how different stimulation modes affected subjects’ donation and SRI behaviors. Neuroscientific studies have found that the rTPJ plays an important role in the psychological mechanism of altruism, especially in the processing of moral-material conflicts (Morishima et al., 2012; Jeurissen et al., 2014; van der Meulen et al., 2016; Obeso et al., 2018; Li et al., 2020). Based on this evidence, we tested the following two hypotheses. First, modulating activity in the rTPJ using tDCS will alter the subjects’ processing of moral-material conflicts, changing the subjects’ donation behaviors. Second and more importantly, if altruism does play an important role in SRI decision-making, changes in the subjects’ processing of moral-material conflicts will also lead to changes in their SRI behaviors.

We conducted four sequential tasks in an experiment. First, we tested whether modulating activity in the rTPJ successfully altered the subjects’ levels of altruism through the use of a donation task. Second, we designed a simulated SRI task and compared the subjects’ willingness to invest in an ordinary asset and their willingness to invest in a socially responsible asset to study whether different stimulation modes changed non-financial motives to engage in SRI. On this basis, we further studied the willingness to invest in a real socially responsible fund under different stimulation modes through the use of a real SRI task. Finally, to control for the subjects’ risk preferences regarding their investment decisions, we measured this variable through the use of a risk preference measurement task. We found that enhancing activity in the rTPJ increased the subjects’ donation amounts while decreasing activity in the rTPJ reduced donation amounts. This result verifies our first hypothesis and is consistent with the conclusions of existing studies (Morishima et al., 2012; Hutcherson et al., 2015; Strombach et al., 2015). More importantly, by observing bids made for the simulated and real socially responsible asset (fund) under different stimulation modes, we found that the subjects who received anodal stimulation also showed a stronger willingness to invest in SRI from non-financial motives. Therefore, our second hypothesis is also verified, and we have reason to believe that altruism does play an important role in SRI decision-making. Increasing activity in the rTPJ effectively reduced the monetary self-interest of the subjects, increasing their willingness to make SRIs.

Nevertheless, we found that a decrease in rTPJ activity did not have a significant impact on SRI behavior. Although the “anodal excitation, cathodal inhibition effect” (AeCi-effect, Jacobson et al., 2012) has been observed in many studies investigating the motor system and other cortical regions, such as the visual cortex (Antal et al., 2003; Lang et al., 2004; Furubayashi et al., 2008; Stagg et al., 2009), a meta-analysis showed that the AeCi-effect has rarely been found in cognitive studies (Jacobson et al., 2012; Brückner and Kammer, 2017). In most cases, anodal stimulation has indeed improved performance, while the effect and direction of modulation caused by cathodal tDCS may depend on the task investigated (Brückner and Kammer, 2017). In our study, cathodal stimulation inhibited the subjects’ behaviors for the donation task but not for the SRI task, which indicates that there may still be some processing differences between charity donation and SRI. To gain more insight, we checked the subjects’ perceived effectiveness of charity donation and socially responsible fund and found that anodal stimulation did not alter the subjects’ perceived effectiveness of both, while cathodal stimulation decreased that of charity donation. Thus, anodal and cathodal stimulations may influence subjects’ donation behaviors through different channels: An increase in rTPJ activity reduced monetary self-interest, while a decrease in rTPJ activity reduced perceived effectiveness. In contrast, in the context of SRI, an increase in rTPJ activity still reduced monetary self-interest, but the perceived effectiveness of socially responsible fund did not change because this may be determined through more rational thinking than that of charity donations.

Our study also draws some other interesting conclusions. For example, we found a significant impact of gender on bids for the socially responsible assets and fund, which can be compared to the evidence of previous studies (Nilsson, 2008; Cheah et al., 2011; Dorfleitner and Utz, 2014). Notably, these studies found female investors to be more willing to make SRIs than male investors, while we found males to be more willing to make SRIs than females. A possible explanation could be that females may be more cautious about new concepts or about engaging in unfamiliar practices such as SRI, which is not yet a well-known investment philosophy in China. In addition, we found that the subjects’ risk preferences significantly affected their investment bids. In line with intuition, the subjects with stronger risk preferences made larger bids for their investments. We also investigated the role of perceived effectiveness in SRI. Perceived effectiveness refers to the fact that people are more likely to take actions when they believe that their actions will help solve certain problems (Straughan and Roberts, 1999). Beal et al. (2005) showed that investors gain psychological value when they feel that they have made contributions to a worthy cause or have done something for others, and this feeling serves as an important impetus for them to make SRIs. Studies have also found a significant positive correlation between perceived effectiveness and the willingness to make SRIs (Nilsson, 2008; Wins and Zwergel, 2016; Brodback et al., 2019). Consistent with the above conclusions, our study shows that the stronger subjects’ perceived effectiveness was, that is, the more they believed that charity donation and SRI could have positive effects on society and the greater their willingness to make SRIs became.

Nevertheless, this work presents some limitations. First, although we balanced the gender ratio across the stimulation conditions, the number of male and female subjects was not the same due to limitations during recruitment. We also ran analyses on male and female subject samples. The results for the female subject sample (72 subjects) are the same as those for the overall sample, while the results for the male subject sample (24 subjects) show the same tendencies but are not significant. The insignificant results of the male subject sample may be due to a gender difference in the function of the TPJ in the processing mechanisms. The latter case is also supported by previous studies showing gender differences in TPJ activation in investment decision-making occurring in trust games and in other social cognitive tasks, with higher activation found in males than in females (Schulte-Rüther et al., 2008; Luo et al., 2015; Lemmers-Jansen et al., 2017, 2019). Nevertheless, the neurobiological and psychosocial factors behind such differences are still unclear and need to be explored by future studies. Second, the stimulation of the rTPJ may also affect several other brain functions. For example, Gerfo et al. (2019) found that anodal tDCS over the rTPJ increased the number of antisocial punishment choices made compared to sham conditions. Wang et al. (2019) found enhanced activity in the rTPJ via anodal stimulation to increase the accuracy of a participant’s inference of the strategies of others or a participant’s concern for others and thus helped a participant bid optimally in a competition context. Although the tasks used in our study did not involve punishment decisions or interactions between subjects, it is still difficult to exclude the possibility that some functions related to the decision-making process involved in the experiment may also have been modified by the stimulations. Furthermore, with the other electrode placed over the subject’s left cheek, the current may also have flowed over somatosensory and left hemisphere parietal/temporal regions. The method used to place electrodes in this study was adopted from previous studies and was used to reduce the impact of the non-target electrode on the brain cortex (Berryhill and Jones, 2012; Tseng et al., 2012; Mai et al., 2016). By placing the non-target electrode on the contralateral cheek instead of on other cortex areas, we tried to reduce the inhibition of activity in other cortex areas. Nevertheless, this is a methodological limitation of our study.

Other limitations may also include the particularities of our subjects. The subjects involved in our study are university students, and these young, smart, and educated subjects may have different underlying psychological/social beliefs that might influence investment behaviors relative to the broader population. In addition, there are differences between asset or fund bids and real-world investment decisions. For instance, to be more consistent with previous studies, we did not test the effect of stimulations on the magnitude of investment, which is a crucial facet of real-world investment decisions.

To summarize, this study used tDCS to temporarily modulate activity in the rTPJ and tested how different stimulation modes affected subjects’ donation and SRI behaviors. We found that anodal stimulation increased the subjects’ donation amounts, while cathodal stimulation decreased their donation amounts. More importantly, we found that anodal stimulation could enhance subjects’ willingness to make SRIs, suggesting that altruism plays an important role in SRI decision-making. Nevertheless, cathodal stimulation did not reduce subjects’ willingness to make SRIs. Furthermore, cathodal stimulation changed subjects’ perceived effectiveness of charitable donation but not that of socially responsible fund. This may help explain the inconsistent effects of cathodal stimulation on charitable donations and SRI behaviors. The main contribution of our study lies in its pioneering application of tDCS to conduct research on SRI behaviors and provision of neuroscientific evidence regarding the role of altruism in SRI decision-making. As our results show that altruism does play an important role in SRI decision-making, practical applications could involve increasing the amount of SRI with methods that can evoke or increase altruism. Our results also imply that increasing and decreasing activity in the rTPJ may lead to different processing mechanisms involved in altruistic tasks.

## Data Availability Statement

The raw data supporting the conclusions of this article will be made available by the authors, without undue reservation.

## Ethics Statement

The studies involving human participants were reviewed and approved by Ethics Committee of the Key Laboratory of Applied Brain and Cognitive Sciences, Shanghai International Studies University. The patients/participants provided their written informed consent to participate in this study.

## Author Contributions

XY, WM, SC, MG, and JZ designed the experiment, revised the manuscript, and approved the version to be published. WM, SC, and MG performed the experiment. XY, WM, and SC analyzed the data and wrote the manuscript. WM and SC created the figures. All authors contributed to the article and approved the submitted version.

## Conflict of Interest

The authors declare that the research was conducted in the absence of any commercial or financial relationships that could be construed as a potential conflict of interest.

## Publisher’s Note

All claims expressed in this article are solely those of the authors and do not necessarily represent those of their affiliated organizations, or those of the publisher, the editors and the reviewers. Any product that may be evaluated in this article, or claim that may be made by its manufacturer, is not guaranteed or endorsed by the publisher.
